# Incidence of Invasive Pneumococcal Disease in US Children by Risk Group, 1998-2023

**DOI:** 10.1093/jpids/piag084

**Published:** 2026-08-29

**Authors:** Salini Mohanty, Nicolae Done, Yan Song, Travis Wang, Abigail Zion, Emily Reichert, Tayler Li, Meghan White, Kelsie Cassell, Yi-Ling Huang, Jessica P Weaver, Kelly D Johnson, Natalie Banniettis, Kristen A Feemster

**Affiliations:** Merck & Co., Inc., Rahway, NJ, United States; Analysis Group, Inc., Boston, MA, United States; Analysis Group, Inc., Boston, MA, United States; Analysis Group, Inc., Boston, MA, United States; Analysis Group, Inc., Boston, MA, United States; Analysis Group, Inc., Boston, MA, United States; Analysis Group, Inc., Boston, MA, United States; Merck & Co., Inc., Rahway, NJ, United States; Merck & Co., Inc., Rahway, NJ, United States; Merck & Co., Inc., Rahway, NJ, United States; Merck & Co., Inc., Rahway, NJ, United States; Merck & Co., Inc., Rahway, NJ, United States; Merck & Co., Inc., Rahway, NJ, United States; Merck & Co., Inc., Rahway, NJ, United States

**Keywords:** children, chronic medical conditions, immunocompromising conditions, incidence rates, invasive pneumococcal disease

## Abstract

**Background:**

Children with risk conditions are vulnerable to invasive pneumococcal disease (IPD) and are targeted for routine and supplemental pneumococcal vaccination. This study aimed to quantify the incidence of IPD in US children by risk group since pneumococcal conjugate vaccine introduction.

**Methods:**

Invasive pneumococcal disease episodes were identified in the Merative™ MarketScan® Commercial Database (1998-2023) and Multi-State Medicaid Database (2001-2023) using inpatient and outpatient claims. Annual IPD incidence rates (IRs) were calculated as episodes per 100 000 person-years (PY) for children <18 years of age, overall and by insurance type, age, and risk group. Incidence rate ratios (IRRs) were calculated to compare IR for children with chronic medical conditions (CMC) and immunocompromising conditions (IC) with those with no CMC/IC.

**Results:**

A total of 3538 commercially-insured and 3268 Medicaid-insured children with ≥1 IPD episode were identified among 128.9 million (121.9 million PYs) and 71.2 million eligible children (65.4 million PYs), respectively. Across age and risk groups, IPD IRs decreased over time, regardless of insurance type. Commercially-insured children with CMC and IC had 3.1-8.6 and 19.1-74.5 times higher IPD IRs, respectively, than those without CMC/IC, with similar patterns in the Medicaid database. Invasive pneumococcal disease IRs were the highest among children <2 years, while risk-group disparities were greater among older children. Incidence rate ratios comparing CMC and IC vs. no CMC/IC increased over time, indicating widening disparities.

**Conclusions:**

Invasive pneumococcal disease IRs among children with CMC/IC remain substantially higher than those without CMC/IC, and IRRs for children with risk conditions have increased over time. The risk-group disparities were more pronounced in older children. Better prevention strategies are needed to prevent IPD in children with risk conditions.

## INTRODUCTION

Invasive pneumococcal disease (IPD) is defined as isolation of *Streptococcus pneumoniae* from a normally sterile site (eg, blood or cerebrospinal fluid). The most common invasive manifestations in children are bacteremic pneumonia, bacteremia without focus, and meningitis. Since 2000, several pneumococcal conjugate vaccines (PCVs) have been licensed for prevention of pneumococcal disease (PD) in infants and children in the United States, including the 7-valent (PCV7) in 2000, 13-valent (PCV13) in 2010, 15-valent (PCV15) in 2022, and 20-valent (PCV20) in 2023.

The inclusion of PCVs in the pediatric vaccination schedule over the past 2 decades has led to dramatic reductions in the incidence of IPD.^1–6^ However, a residual burden of IPD persists in the pediatric population, with a slower decline in IPD incidence observed in the late PCV13 period,^7^ due to serotype replacement and breakthrough infections with vaccine-type serotypes. This residual burden is particularly pronounced among children with risk conditions, compared to children without risk conditions.^8–11^ Children with immunocompromising conditions (IC) are predisposed to IPD due to weakened innate and adaptive immunity, including reduced antibody production, neutrophil and complement function, and/or splenic clearance, which limits children’s ability to clear nasopharyngeal pneumococcal colonization and prevent bacterial invasion.^12^^,^^13^ Certain chronic medical conditions (CMC), including chronic lung, liver, and kidney disease, also increase IPD risk. For example, asthma impairs mucociliary clearance and airway defenses, and corticosteroid use further weakens immune control, increasing colonization, pneumonia risk, and likelihood of invasive infection.^11^

The US Centers for Disease Control and Prevention’s (CDC) Advisory Committee on Immunization Practices (ACIP) has provided risk-based pneumococcal vaccine recommendations for children since the early 2000s, with guidance evolving over time to expand protection for children with risk conditions.^14–18^ Current recommendations for all children aged 2-23 months include either PCV15 or PCV20 at 2, 4, 6, and 12-15 months. For children from 2 to 18 years with risk conditions, a dose of PCV20 or ≥1 dose of 23-valent pneumococcal polysaccharide vaccine (PPSV23) is recommended, unless ≥1 dose of PCV20 was administered in the primary series.^19^^,^^20^

Prior studies have highlighted the elevated risk of IPD among children with risk conditions, but most studies focused on individual risk conditions.^11–13^^,^^21^^,^^22^ Data on disease incidence among children with risk conditions during the post-PCV13 period remained limited. To address these gaps, this study quantified the incidence rates (IRs) of IPD and its manifestations among both commercially and Medicaid-insured children in the US to provide a more up-to-date and comprehensive assessment of IPD burden by risk group.

## METHODS

### Data Source

This study used data from the Merative™ MarketScan® Commercial Database (1998-2023) and Multi-State Medicaid Database (2001-2023). The Commercial Database captures data for over 273 million unique enrollees covered by employer-sponsored private health insurance from approximately 350 payers across the United States.^23^ The Medicaid Database comprises data for Medicaid enrollees from 7 to 12 geographically diverse states.^23^ States included in the Medicaid Database are not publicly available, and detailed information on eligibility and population served is not disclosed. The study used de-identified secondary data and was considered exempt research under 45 CFR § 46.104(d)(4) and in compliance with the Health Insurance Portability and Accountability Act, specifically, 45 CFR § 164.514.

### Study Design and Patient Population

This retrospective observational cohort study included children <18 years of age who were enrolled in commercial or Medicaid plans for a continuous calendar year; partial year data were utilized for birth year and 18th birthday year. The study period was divided into 7 periods: (1) Pre-PCV7 (1998-1999, commercial only); (2) Early PCV7 (2001-2005); (3) Late PCV7 (2006-2009); (4) Early PCV13 (2011-2013); (5) Late PCV13 (2014-2019); (6) COVID-19 (2020-2021); and (7) Post-COVID-19 (2022-2023). The years 2000 and 2010, the years that PCV7 and PCV13 were introduced, were considered transitional years.

### Study Outcomes

#### Invasive Pneumococcal Disease Episodes

Invasive pneumococcal disease episodes were identified in each calendar year following the same approach in Hu et al. 2022,^3^ which used International Classification of Diseases 9/10th Revision, Clinical Modification (ICD-9-CM and ICD-10-CM) codes in the inpatient or outpatient claims, a 90-day gap, and a hierarchy of IPD manifestations (meningitis > bacteremia > bacteremic pneumonia > other IPD (eg, arthritis, peritonitis, pericarditis, endocarditis, and osteomyelitis) to avoid double-counting. The specific diagnosis codes are shown in Supplementary Table S1.

A sensitivity analysis was conducted restricting to IPD episodes with ≥1 inpatient admission. This approach assessed the robustness of the findings of the main analysis, as the claims-based algorithm for constructing IPD episodes may have captured follow-up encounters rather than the initial hospitalization at which IPD was first diagnosed.

#### Risk Group and Age Stratification

Centers for Disease Control and Prevention ACIP-defined risk conditions for PD were identified annually using diagnosis and procedure codes (see Supplementary Table S2). All available data from the birth date or start of the data period were used to identify the earliest diagnosis of eligible risk conditions, which was then carried forward to all subsequent calendar years. For all years following the year of the earliest observed diagnosis of a risk condition, risk condition status was therefore established prior to the identification of any IPD episode; in the calendar year in which the earliest diagnosis of a risk condition was observed, the risk condition may have been documented before, during, or after an IPD episode. Children were further categorized into 1 of 3 mutually exclusive risk groups within each calendar year, based on the CDC ACIP risk-based recommendations for pneumococcal vaccination in children: (1) IC ± CMC: children with IC, with or without CMC, (2) CMC: children with CMC but no IC, and (3) no CMC/IC: children without risk conditions. Immunocompromising conditions included congenital or acquired asplenia or splenic dysfunction, congenital or acquired immunodeficiency, diseases or conditions treated with immunosuppressive drugs or radiation therapy, HIV infection, sickle cell disease and other hemoglobinopathies, solid organ transplant, and maintenance dialysis or nephrotic syndrome. Chronic medical conditions included chronic heart, liver, kidney, or lung disease (including asthma), diabetes, a cerebrospinal fluid leak, or a cochlear implant.^20^ Age was divided into the following categories: <2 years, 2-4 years, and 5-17 years. For children with enrollment data in their birth year, the date of birth was imputed as the 15th day of the first enrollment month; for others, July 1 of the birth year was used.

### Statistical Analysis

#### Analyses of Incidence Rates

Annual IRs for overall and specific IPD manifestations were calculated as episodes per 100 000 person-years (PY), using the total number of episodes divided by the total PY of health plan enrollment in each calendar year. For each of the 7 time periods, IRs were calculated as the average of annual IRs within that period. Annual IRs were further stratified by age and risk group for commercially and Medicaid-insured population. Unadjusted incidence rate ratios (IRRs) were calculated as the IR in children with IC ± CMC or CMC divided by that in the children without CMC/IC. Ninety-five percent confidence intervals were calculated using the log-Poisson (Wald) method. Statistical analyses were conducted using SAS version 9.4 (SAS Institute, Inc., Cary, NC).

## RESULTS

### Overall Study Population

Throughout the study period, an average of 4.96 million commercially-insured children contributed 4.69 million PY annually, and an average of 3.10 million Medicaid-insured children contributed 2.84 million PY each year. The demographic characteristics of the overall study populations are summarized by PD risk group in Supplementary Table S3.

Among commercially-insured children, 11.3% had CMC (no IC) and 0.5% had IC ± CMC, compared with 22.1% and 1.0%, respectively, among Medicaid-insured children (Supplementary Table S3). In both populations, the most common CMC was chronic lung disease, particularly asthma (Medicaid: 15.0%; commercial: 7.7%). The most common IC was congenital or acquired immunodeficiencies in commercially-insured and sickle cell disease or other hemoglobinopathy in Medicaid-insured children. The risk conditions in the study populations are summarized by risk group in Supplementary Table S3.

### Population with IPD Episodes

Demographic characteristics for children with IPD episodes, stratified by payer and risk group are presented in Table 1. The mean (SD) age was 5.4 (5.2) years among commercially-insured and 4.5 (4.8) years among Medicaid-insured children. Approximately 41% of commercially-insured and 32% of Medicaid-insured children with IPD were aged 5–17 years. Mean age increased by risk group and was highest among children with IC among both insurance types.

**Table 1:** Sociodemographic characteristics^a^ and risk conditions for the commercially and Medicaid insured patients with IPD episodes (1998-2023), by payer and risk group

	Commercially insured (1998-2023)				Medicaid insured (2001-2023)			
	Overall	No CMC/IC	CMC (no IC)	IC ± CMC	Overall	No CMC/IC	CMC (no IC)	IC ± CMC
**Total number of patients with IPD episode, *N***	3538	2108	956	474	3268	1449	1171	648
**Age** ^**b**^ **, mean (SD)**	5.4 (5.2)	4.8 (5.2)	6.0 (5.1)	7.0 (5.1)	4.5 (4.8)	3.2 (4.2)	4.8 (4.9)	6.6 (4.9)
<2 years, *n* (%)	1365 (38.6%)	997 (47.3%)	284 (29.7%)	84 (17.7%)	1512 (46.3%)	891 (61.5%)	489 (41.8%)	132 (20.4%)
2-4 years, *n* (%)	733 (20.7%)	377 (17.9%)	225 (23.5%)	131 (27.6%)	707 (21.6%)	264 (18.2%)	275 (23.5%)	168 (25.9%)
5-17 years, *n* (%)	1440 (40.7%)	734 (34.8%)	447 (46.8%)	259 (54.6%)	1049 (32.1%)	294 (20.3%)	407 (34.8%)	348 (53.7%)
**Female, *n* (%)**	1619 (45.8%)	993 (47.1%)	421 (44.0%)	205 (43.2%)	1440 (44.1%)	670 (46.2%)	494 (42.2%)	276 (42.6%)
**Region, *n* (%)**								
Northeast	469 (13.3%)	279 (13.2%)	125 (13.1%)	65 (13.7%)	–	–	–	–
North Central	828 (23.4%)	496 (23.5%)	208 (21.8%)	124 (26.2%)	–	–	–	–
South	1504 (42.5%)	867 (41.1%)	417 (43.6%)	220 (46.4%)	–	–	–	–
West	682 (19.3%)	427 (20.3%)	194 (20.3%)	61 (12.9%)	–	–	–	–
Missing/unknown	55 (1.6%)	39 (1.9%)	12 (1.3%)	4 (0.8%)	–	–	–	–
**Urbanicity, *n* (%)**								
Urban	2829 (80.0%)	1669 (79.2%)	763 (79.8%)	397 (83.8%)	–	–	–	–
Rural	580 (16.4%)	377 (17.9%)	148 (15.5%)	55 (11.6%)	–	–	–	–
Missing/unknown	129 (3.6%)	62 (2.9%)	45 (4.7%)	22 (4.6%)	–	–	–	–
**Race and ethnicity, *n* (%)**								
White	–	–	–	–	1337 (40.9%)	637 (44.0%)	511 (43.6%)	189 (29.2%)
Black	–	–	–	–	1073 (32.8%)	486 (33.5%)	366 (31.3%)	221 (34.1%)
Hispanic	–	–	–	–	221 (6.8%)	102 (7.0%)	81 (6.9%)	38 (5.9%)
Other	–	–	–	–	483 (14.8%)	184 (12.7%)	151 (12.9%)	148 (22.8%)
Missing/unknown	–	–	–	–	154 (4.7%)	40 (2.8%)	62 (5.3%)	52 (8.0%)
**Risk conditions, *n* (%)**								
CMCs								
Chronic lung disease	977 (27.6%)	–	776 (81.2%)	201 (42.4%)	1413 (43.2%)	–	1030 (88.0%)	383 (59.1%)
Asthma	568 (16.1%)	–	453 (47.4%)	115 (24.3%)	828 (25.3%)	–	604 (51.6%)	224 (34.6%)
Chronic heart disease	394 (11.1%)	–	227 (23.7%)	167 (35.2%)	622 (19.0%)	–	351 (30.0%)	271 (41.8%)
Diabetes mellitus	48 (1.4%)	–	21 (2.2%)	27 (5.7%)	43 (1.3%)	–	19 (1.6%)	24 (3.7%)
Chronic kidney disease	117 (3.3%)	–	39 (4.1%)	78 (16.5%)	142 (4.3%)	–	47 (4.0%)	95 (14.7%)
Chronic liver disease	90 (2.5%)	–	24 (2.5%)	66 (13.9%)	135 (4.1%)	–	38 (3.2%)	97 (15.0%)
Cochlear implant	54 (1.5%)	–	41 (4.3%)	13 (2.7%)	30 (0.9%)	–	29 (2.5%)	1 (0.2%)
Cerebrospinal fluid leak	38 (1.1%)	–	31 (3.2%)	7 (1.5%)	45 (1.4%)	–	34 (2.9%)	11 (1.7%)
ICs								
Congenital/acquired immunodeficiencies	323 (9.1%)	–	0 (0.0%)	323 (68.1%)	357 (10.9%)	–	0 (0.0%)	357 (55.1%)
Diseases/conditions treated with immunosuppressive drugs/radiation therapy	178 (5.0%)	–	0 (0.0%)	178 (37.6%)	183 (5.6%)	–	0 (0.0%)	183 (28.2%)
Sickle cell disease/other hemoglobinopathies	163 (4.6%)	–	0 (0.0%)	163 (34.4%)	328 (10.0%)	–	0 (0.0%)	328 (50.6%)
Solid organ transplant	114 (3.2%)	–	0 (0.0%)	114 (24.1%)	151 (4.6%)	–	0 (0.0%)	151 (23.3%)
Maintenance dialysis/with nephrotic syndrome	38 (1.1%)	–	0 (0.0%)	38 (8.0%)	56 (1.7%)	–	0 (0.0%)	56 (8.6%)
Congenital/acquired asplenia/splenic dysfunction	36 (1.0%)	–	0 (0.0%)	36 (7.6%)	81 (2.5%)	–	0 (0.0%)	81 (12.5%)
HIV infection	4 (0.1%)	–	0 (0.0%)	4 (0.8%)	13 (0.4%)	–	0 (0.0%)	13 (2.0%)

Abbreviations: CMC, chronic medical condition; COBRA, Consolidated Omnibus Budget Reconciliation Act; HIV, human immunodeficiency virus; IC, immunocompromising condition; IPD, invasive pneumococcal disease; SD, standard deviation; SSA, Social Security Act.

^a^Children’s demographic characteristics were determined for each calendar year. If a child had multiple unique values for the same category in a calendar year, the non-missing value that was observed for the longest time period was selected. If there were ties, the demographic characteristic that came first chronologically was selected.

^b^For children with health plan enrollment data available in their birth year, the date of birth was imputed as the 15th of the first month of health plan enrollment. For all other children, age was calculated using an imputed date of birth of July 1 of the birth year.

Children with an IPD episode were more likely to have a risk condition, compared to the overall study population. Among commercially-insured children with ≥1 episode of IPD, 27.0% had a CMC (no IC) and 13.4% had an IC ± CMC, compared with 35.8% and 19.8%, respectively, among Medicaid-insured children with ≥1 episode of IPD.

The distribution of risk conditions for children with IPD episodes is summarized in Table 1, by payer and risk group. The most common risk conditions were similar across insurance types. For children with CMC (no IC), the most common CMCs were chronic lung disease (commercial: 81.2%; Medicaid: 88.0%), which included asthma (47.4%; 51.6%), and chronic heart disease (23.7%; 30.0%). Among children with IC ± CMC, common ICs included congenital or acquired immunodeficiencies (68.1%; 55.1%), diseases/conditions treated with immunosuppressive medications or radiation therapy (37.6%; 28.2%), and sickle cell disease or other hemoglobinopathies (34.4%, 50.6%). Chronic medical conditions were also common in children with IC, including chronic lung disease in 42.4% of commercially-insured and 59.1% of Medicaid-insured children with IC (see Table 1).

### Incidence Rates of Invasive Pneumococcal Disease

#### Overall Invasive Pneumococcal Disease

**Figure 1:**
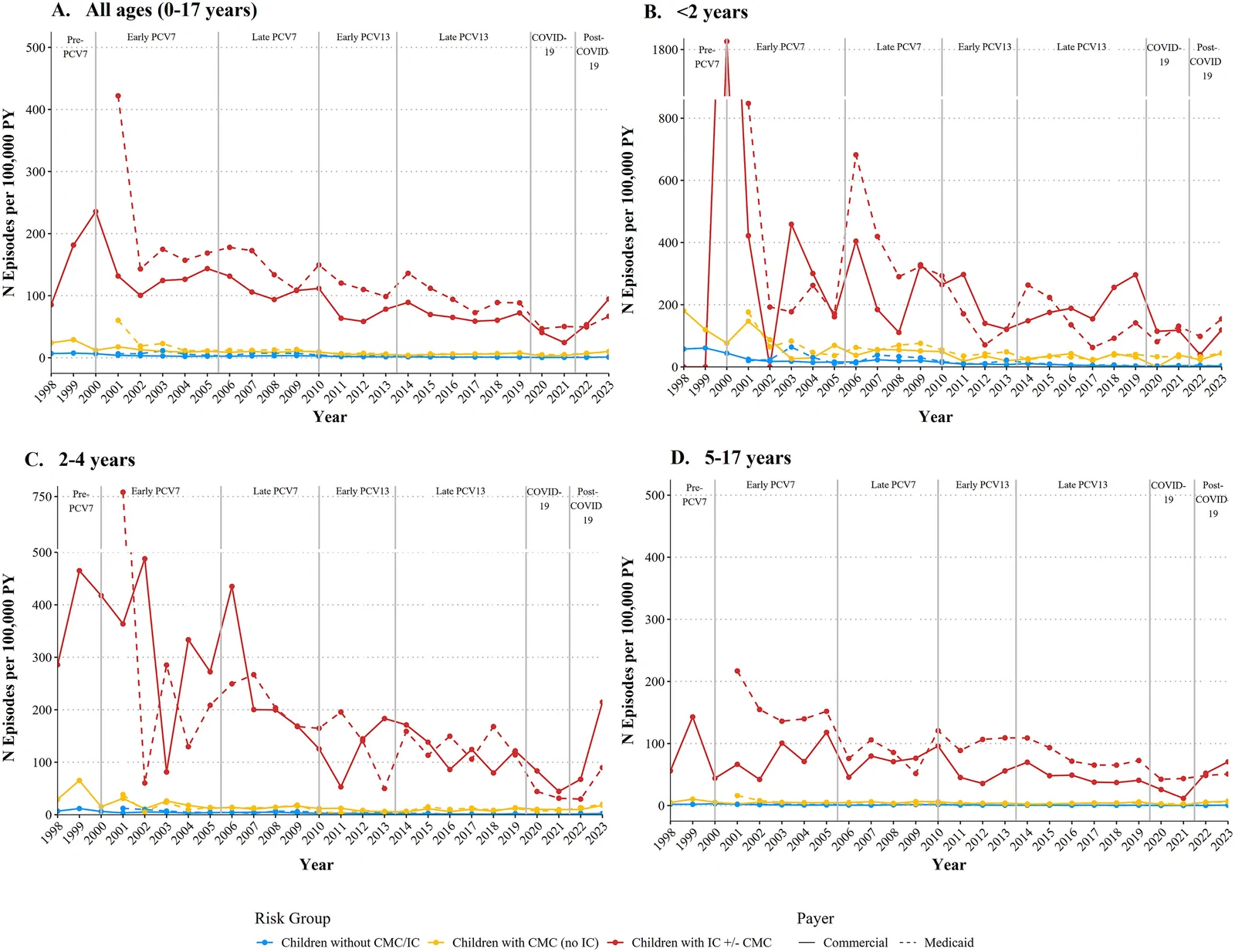
Trends in Annual Overall IPD Episode IRs among Commercially and Medicaid Insured Children (1998-2023), by Age, Payer, and Risk group^a^. Abbreviations: CMC, chronic medical condition; COVID-19, coronavirus disease 2019; IC, immunocompromising condition; IPD, invasive pneumococcal disease; IR, incidence rate; PCV, pneumococcal conjugate vaccine; PY, person-year. ^a^Panels B and C have both y-axis breaks and higher y-axis maximum values than panels A and D, due to multiple years with extreme IR values >500 episodes per 100 000 PY for children with IC <2 years and 2-4 years.

Annual IRs for IPD episodes are presented graphically by age, payer, and risk group in Fig. 1. Across all strata and age groups, IPD IRs decreased over time in both databases, with children <2 years generally showing the highest rates. Children with CMC/IC had substantially higher IPD IRs than those without CMC/IC, regardless of insurance type, with the highest IRs in children with IC ± CMC. Medicaid-insured children had generally higher IPD IRs than commercially-insured children, although the differences narrowed over time.

Annual IRs for overall IPD by PCV period, age group, and risk group are shown in Table 2 for both commercially insured and Medicaid-insured children. In both databases, the overall IPD IRs declined within each risk group from the earliest available PCV period to their lowest in the COVID-19 period, then partially rebounded in the post-COVID-19 period.

**Table 2:** Incidence rates and rate ratios of IPD among commercially (1998-2023) and Medicaid-insured (2001-2023) children, by PCV period, age, and risk group

Period	All ages				<2 years				2-4 years				5-17 years			
	Overall	No CMC/IC	CMC (no IC)	IC ± CMC	Overall	No CMC/IC	CMC (no IC)	IC ± CMC ^a^	Overall	No CMC/IC	CMC (no IC)	IC ± CMC ^a^	Overall	No CMC/IC	CMC (no IC)	IC ± CMC
**Commercially insured children**																
**Incidence rates (per 100 000 PYs)** ^b^																
Pre PCV7	9.47	7.18	26.84	137.34	67.74	59.24	148.43	0.00	16.21	9.37	48.94	384.62	2.98	2.11	8.02	102.91
Early PCV7	3.86	2.75	10.95	128.83	20.93	16.79	57.27	254.60	6.41	3.98	17.67	287.79	1.69	1.13	4.60	87.83
Late PCV7	4.17	3.08	9.64	108.64	23.17	19.84	50.17	255.50	6.34	4.19	13.96	236.42	1.83	1.13	5.37	69.54
Early PCV13	2.48	1.76	5.40	66.47	10.55	8.62	24.59	186.96	3.56	2.23	8.48	126.87	1.48	0.95	3.62	45.27
Late PCV13	2.26	1.20	5.64	69.96	9.60	6.34	32.54	204.33	3.04	1.37	8.43	121.54	1.35	0.58	3.68	47.92
COVID-19	1.16	0.54	3.24	32.76	4.92	2.99	18.69	116.17	2.21	0.72	10.10	64.52	0.56	0.21	1.55	18.97
Post COVID-19	2.57	1.00	8.56	74.30	6.17	3.36	33.34	79.13	4.47	1.95	13.85	142.81	1.84	0.54	6.26	61.45
**IRRs (vs. no risk conditions) [95% CI]** ^c^																
Pre PCV7	–	–	3.74 [2.49, 5.62]	19.14 [8.86, 41.37]	–	–	2.51 [1.39, 4.53]	1.95 [0.12, 31.57]	–	–	5.22 [2.37, 11.51]	41.06 [11.80, 142.88]	–	–	3.80 [1.61, 8.99]	48.83 [16.69, 142.86]
Early PCV7	–	–	3.98 [3.27, 4.85]	46.88 [35.68, 61.61]	–	–	3.41 [2.52, 4.62]	15.16 [7.76, 29.63]	–	–	4.44 [3.01, 6.57]	72.35 [43.61, 120.03]	–	–	4.06 [2.86, 5.76]	77.55 [52.47, 114.61]
Late PCV7	–	–	3.13 [2.71, 3.61]	35.25 [28.76, 43.20]	–	–	2.53 [2.00, 3.21]	12.88 [8.02, 20.67]	–	–	3.33 [2.46, 4.51]	56.48 [39.08, 81.62]	–	–	4.76 [3.77, 6.02]	61.69 [45.56, 83.52]
Early PCV13	–	–	3.08 [2.56, 3.69]	37.87 [29.79, 48.15]	–	–	2.85 [2.00, 4.06]	21.69 [12.78, 36.82]	–	–	3.81 [2.56, 5.66]	56.89 [35.29, 91.71]	–	–	3.82 [2.95, 4.94]	47.70 [34.13, 66.69]
Late PCV13	–	–	4.71 [3.98, 5.58]	58.48 [48.08, 71.11]	–	–	5.13 [3.83, 6.88]	32.23 [21.99, 47.24]	–	–	6.17 [4.11, 9.26]	88.99 [58.09, 136.35]	–	–	6.34 [4.93, 8.15]	82.69 [62.19, 109.95]
COVID-19	–	–	6.04 [3.75, 9.72]	61.06 [35.93, 103.78]	–	–	6.26 [2.72, 14.40]	38.90 [15.44, 97.99]	–	–	13.94 [5.40, 35.95]	89.03 [29.92, 264.92]	–	–	7.26 [3.25, 16.22]	88.71 [37.68, 208.88]
Post COVID-19	–	–	8.59 [6.13, 12.03]	74.54 [51.13, 108.65]	–	–	9.93 [5.04, 19.54]	23.56 [8.02, 69.26]	–	–	7.12 [3.51, 14.44]	73.39 [35.65, 151.10]	–	–	11.52 [7.11, 18.67]	112.99 [67.09, 190.28]
**Medicaid-insured children**																
**Incidence rates (per 100 000 PYs)** ^b^																
Early PCV7	10.09	6.86	17.69	183.60	39.10	30.58	67.68	254.13	10.66	6.83	15.21	220.83	3.59	1.88	5.79	151.17
Late PCV7	9.23	6.48	12.75	144.14	38.04	28.81	66.49	403.91	9.75	5.06	14.96	216.30	2.76	1.53	3.67	78.08
Early PCV13	5.05	3.12	6.77	108.47	19.73	14.43	42.27	117.88	4.98	3.17	5.65	122.75	2.51	0.79	3.54	102.74
Late PCV13	3.65	1.58	6.09	96.77	11.47	6.89	31.13	145.96	5.00	1.55	10.80	133.81	2.14	0.63	3.38	78.06
COVID-19	2.28	0.77	5.01	48.54	7.83	3.61	32.84	104.31	2.42	0.84	7.41	38.26	1.44	0.26	2.97	42.98
Post COVID-19	3.50	1.29	8.18	56.62	9.21	4.28	37.24	120.03	4.64	1.77	16.04	53.67	2.48	0.70	5.39	49.45
**IRRs (vs. no risk conditions) [95% CI]** ^c^																
Early PCV7	–	–	2.58 [2.19, 3.03]	26.75 [21.78, 32.86]	–	–	2.21 [1.79, 2.73]	8.31 [5.56, 12.43]	–	–	2.23 [1.57, 3.16]	32.32 [21.35, 48.92]	–	–	3.07 [2.12, 4.47]	80.23 [57.22, 112.50]
Late PCV7	–	–	1.97 [1.66, 2.33]	22.23 [17.73, 27.88]	–	–	2.31 [1.86, 2.87]	14.02 [9.65, 20.37]	–	–	2.96 [2.04, 4.29]	42.77 [27.15, 67.36]	–	–	2.40 [1.61, 3.58]	51.09 [33.44, 78.05]
Early PCV13	–	–	2.17 [1.76, 2.68]	34.77 [26.98, 44.82]	–	–	2.93 [2.18, 3.93]	8.17 [4.29, 15.56]	–	–	1.78 [1.08, 2.94]	38.68 [22.56, 66.31]	–	–	4.47 [2.92, 6.85]	129.83 [84.45, 199.61]
Late PCV13	–	–	3.87 [3.33, 4.49]	61.38 [52.22, 72.15]	–	–	4.52 [3.56, 5.74]	21.19 [15.39, 29.18]	–	–	6.97 [5.02, 9.67]	86.39 [61.05, 122.24]	–	–	5.35 [4.13, 6.92]	123.59 [95.08, 160.65]
COVID-19	–	–	6.53 [4.64, 9.19]	63.23 [43.05, 92.88]	–	–	9.10 [5.46, 15.16]	28.90 [14.75, 56.63]	–	–	8.86 [4.06, 19.36]	45.78 [18.07, 116.01]	–	–	11.49 [6.08, 21.73]	166.33 [85.91, 322.04]
Post COVID-19	–	–	6.34 [4.70, 8.54]	43.90 [30.59, 62.99]	–	–	8.70 [5.03, 15.07]	28.05 [13.47, 58.40]	–	–	9.04 [4.93, 16.59]	30.25 [13.48, 67.86]	–	–	7.74 [4.91, 12.19]	70.93 [42.42, 118.59]

Abbreviations: CI, confidence interval; CMC, chronic medical condition; COVID-19, coronavirus disease 2019; IC, immunocompromising condition; IPD, invasive pneumococcal disease; IR, incidence rates; IRR, incidence rate ratio; PCV, pneumococcal conjugate vaccine; PY, person-year.

^a^These IR and IRR estimates should be interpreted with caution as the sample size of children aged <2 years and 2-4 years with IC may be insufficient to estimate the IRs of IPD episodes with adequate precision. In particular, the total PYs during the pre PCV7 period for children aged <2 years and 2-4 years with IC were <1000, which may limit the stability and precision of the IR estimates.

^b^IRs for each PCV/COVID-19 period were calculated as the average of annual IRs across the years within that period.

^c^Ninety-five percent CIs for IRRs were estimated using the log-Poisson (Wald) method. For strata with zero events, a 0.5 continuity correction was applied when calculating the IRR and 95% CIs to enable estimation of uncertainty. These estimates should be interpreted with caution due to sparse data.

#### Specific Manifestations of Invasive Pneumococcal Disease

For commercially-insured children with CMC (no IC), and no CMC/IC, the incidence of bacteremia, meningitis, bacteremic pneumonia, and other IPD declined over time, with similar patterns observed in the COVID-19 and post-COVID-19-period (see Supplementary Table S4). For children with IC, the IRs of bacteremic pneumonia and other IPD decreased over time, while bacteremia and meningitis IRs showed modest declines and remained elevated, with a decrease in the COVID-19 period and a rebound in the post-COVID-19 period. Trends in the Medicaid population (see Supplementary Table S5) were similar to the commercially-insured population, with few IPD cases among children with IC.

Annual IRs of overall IPD and its manifestations are reported numerically by age and risk group for commercially and Medicaid-insured children in Supplementary Tables S6 and S7, respectively, and stratified by PCV period in Supplementary Tables S8 and S9.

#### Incidence Rate Ratios

Unadjusted average IRRs for overall IPD in children with risk conditions, relative to children with no CMC/IC, are presented by payer and age group over the study period in Table 3. On average, across age groups, children with CMC (no IC) experienced approximately 3-4 times higher IPD IRs than those without CMC/IC (commercial: IRR = 3.6, Medicaid: IRR = 2.8), while children with IC ± CMC had roughly 34-39 times higher IRs (commercial: IRR = 38.7, Medicaid: IRR = 34.2). The relative burden of IPD in children with CMC/IC compared to children without CMC/IC was greater for children aged ≥2 years. Among commercially-insured children with IC ± CMC, overall IPD IRRs versus no CMC/IC were 59.8 (5-17 years), 57.9 (2-4 years) and 17.1 (<2 years), respectively. Among Medicaid-insured children, these IRRs were 89.6, 44.2, and 13.0, respectively. Children with CMC (no IC) showed a similar trend in both populations, with smaller differences between age groups.

**Table 3:** Incidence rate ratios comparing average IR of overall IPD over the follow-up period among children with risk conditions versus children with no risk conditions, by payer and age

Database (period)	Risk group comparison	Average IRR [95% CI] of overall IPD ^a^			
		All ages	Ages <2 years	Ages 2-4 years	Ages 5-17 years
Commercial (1998-2023)	IC ± CMC vs. no CMC/IC	38.65 [35.12, 42.54]	17.05 [13.81, 21.06]	57.87 [47.80, 70.05]	59.80 [52.13, 68.60]
	CMC (no IC) vs. no CMC/IC	3.55 [3.29, 3.82]	3.43 [3.02, 3.90]	4.45 [3.78, 5.23]	4.65 [4.15, 5.21]
Medicaid (2001-2023)	IC ± CMC vs. no CMC/IC	34.21 [31.28, 37.41]	13.03 [10.93, 15.54]	44.16 [36.56, 53.34]	89.58 [77.14, 104.04]
	CMC (no IC) vs. no CMC/IC	2.79 [2.58, 3.00]	3.43 [3.08, 3.82]	3.81 [3.23, 4.50]	4.28 [3.70, 4.95]

Abbreviations: CI, confidence interval; CMC, chronic medical condition; IC, immunocompromising condition; IPD, invasive pneumococcal disease; IR, incidence rates; IRR, incidence rate ratio.

^a^Ninety-five percent CIs for IRRs were estimated using the log-Poisson (Wald) method.

The relative burden of IPD for children with risk conditions increased over time in both commercially and Medicaid-insured populations. Among commercially-insured children, IRRs comparing IC ± CMC and CMC (no IC) with no CMC/IC increased from 19.1 and 3.7 (pre-PCV7) to 74.5 and 8.6 (post-COVID-19) (Table 2). For Medicaid-insured children, the corresponding IRRs increased from 26.8 and 2.6 (early post-PCV7) to 43.9 and 6.3 (post-COVID-19) (Table 2). The same trend was observed for specific IPD manifestations (Supplementary Tables S4 and S5).

#### Inpatient Invasive Pneumococcal Disease (Sensitivity Analysis)

Sensitivity analyses restricted to inpatient IPD episodes found lower absolute IRs, with similar age- and risk-dependent trends in both commercially and Medicaid-insured patients (see Supplementary Fig. S1 and Supplementary Tables S10-S15).

## DISCUSSION

This 25-year longitudinal analysis of trends in the annual incidence of IPD demonstrates a consistent reduction in IPD IRs across age and risk groups following introduction of PCV, consistent with previous literature.^1–4^ However, while substantial reductions were observed across risk groups, children with CMC/IC continue to experience a higher incidence of IPD. Most concerning, the increasing IRRs over time comparing children with and without risk conditions indicate a widening disparity in IPD risk, particularly among children ≥2 years, despite risk-based PCV vaccination guidance in place for this age group.^20^

In the overall study population, approximately 12% of commercially-insured children and 23% of Medicaid-insured children had at least one risk condition. These estimates align with CDC reports and pediatric studies.^24–26^ Notably, asthma was present in 7.7% of commercially-insured and 15.0% of Medicaid-insured children, consistent with a prior report of 7.3%–9.5% overall and up to 18% in socioeconomically disadvantaged children.^27^ Among children with IPD episodes, around half had at least one risk condition, with a higher proportion in Medicaid (55.7%) versus commercially-insured children (40.4%). A substantial proportion of children with IC also had at least one CMC. These numbers are consistent with published estimates from other studies. A Massachusetts surveillance program reported 25.9% of children with IPD had at least one risk condition.^28^ A systematic review found 42.9% of IPD cases with breakthrough infections occurred in children with at least one risk condition.^29^ Estimates may vary due to differences in study methodology, data sources, study year, and the risk conditions identified.

This study assesses IPD risk in children across age and risk groups from the pre-PCV7 to post-COVID-19 era. The study’s findings reinforce the growing body of evidence of higher IPD burden in children with risk conditions.^2^^,^^6^^,^^11–13^^,^^21^ Two recent studies using the CDC’s Active Bacterial Core surveillance data highlighted substantial increases in IPD incidence among children with IC.^12^^,^^13^ Hamilton et al. reported the IRRs for IPD comparing children with versus without hematologic malignancies were 215.8 and 240.9 during the early- and late-PCV13 periods, respectively.^13^ Payne et al. reported that among Black or African American children, the IPD IRR comparing those with versus without sickle cell disease increased from 28 to 42 from the pre-PCV13 to the late-PCV13 periods, and that the IRR was higher in children aged 5-17 years than those aged <5 years.^12^ Similarly, a claims-based cohort with a similar design from the PCV7 period also found that the rate ratios were higher in children aged 5-17 years compared to those <5 years.^5^ These findings are consistent with our results of widening IRRs over time and the greater disparities in IPD rate among older children with risk conditions compared to those without.

Results demonstrate a persistently elevated IPD burden in both commercially and Medicaid-insured children with risk conditions throughout the entire study period, consistent with other published studies.^2^^,^^12^^,^^13^ Despite differences in the prevalence of risk conditions between the insurance populations, the IRRs comparing children with IC ± CMC or CMC versus no CMC/IC showed similar patterns, with both populations exhibiting a widening trend over time.

However, under current ACIP risk-based recommendations, children who receive the full PCV20 3 + 1 series in infancy are not recommended to receive any additional pneumococcal vaccine doses between the 2 and 17 years even if they subsequently develop a risk condition after the age of 2 years. Given that older children with risk conditions continue to experience substantially higher IPD rates than those without CMC/IC and the low vaccine coverage rates among this population, ongoing monitoring and improved implementation of vaccine recommendations beyond the routine series are warranted to provide broader serotype coverage.^20^ Vaccine coverage among children with high-risk conditions remains suboptimal, with reported PPSV23 vaccination rates as low as approximately 20% among eligible high-risk children,^30^ and a substantial proportion of residual pediatric IPD is attributable to serotypes covered by higher-valency vaccines (eg, PCV20 and PCV21).^13^^,^^31^ These findings suggest that both improved uptake of currently recommended vaccines and broader serotype coverage may help reduce the remaining burden. Notably, in June 2026, PCV21 was approved for children and adolescents aged 2–17 years at increased risk for PD, supported in part by findings from the Phase 3 STRIDE-13 trial, and may provide broader protection against residual IPD burden in this population.^32^^,^^33^ Additionally, the incidence of IPD decreased substantially during the COVID-19-period but partially rebounded during the post-COVID period, underscoring the importance of continued surveillance to guide future recommendations.

Strengths of this study include the use of 2 large longitudinal datasets spanning over 2 decades, enabling assessment of IPD incidence trends across PCV eras. These findings provide updated post-PCV13 data on IPD burden among children with risk conditions that reflect updated ACIP recommendations, offering timely evidence for future risk-based immunization strategies. Inclusion of both commercially and Medicaid-insured populations captures a more socioeconomically diverse pediatric population than most prior analyses.

The study has several limitations. First, as with any claims-based analysis, miscoding may lead to misclassification and measurement error. In addition, IPD and its manifestations were identified using diagnosis codes, without lab results and medical charts to verify diagnoses or causative pathogens. Therefore, IRs may be underestimated and differ from surveillance-based estimates. Furthermore, vaccination histories and pneumococcal serotypes for infection episodes were not available, precluding assessment of the role of vaccine uptake and serotype distribution in IPD incidence patterns. Since continuous eligibility was only required in the calendar year of analysis, the true earliest diagnoses of risk conditions may be missed due to left truncation of data. For the calendar year in which an IC or CMC condition was first documented, it was not always possible to determine whether the condition was identified before or after an IPD episode, which may have resulted in some IPD episodes being attributed to the IC or CMC group before the risk condition was documented. Moreover, the transition from ICD-9 to ICD-10 diagnosis code systems in 2015 may have affected the disease classification over time. Additionally, the MarketScan Commercial Database is a non-random sample that underrepresents children insured through small or medium employers and the MarketScan Multi-State Medicaid Database only includes selected states.^23^ Therefore, findings may not be fully generalizable to all US children with insurance. Uninsured children were excluded from the analyses, though they only represent 5% of the pediatric population.^34^ Finally, the IC group is clinically heterogeneous, comprising conditions such as asplenia or splenic dysfunction, congenital or acquired immunodeficiency, immunosuppressive therapy, HIV infection, and hemoglobinopathies; children with asplenia-related conditions may differ from those with other ICs with respect to underlying pneumococcal risk and the use of antimicrobial prophylaxis, which could influence observed IPD risk estimates beyond the effects of vaccination alone. However, antimicrobial prophylaxis was not captured in the data source. These conditions were grouped together to align with the CDC ACIP risk-based vaccination categories that framed this analysis.

## CONCLUSION

Although IPD incidence has decreased since the introduction of PCVs across all risk groups, children with CMC and IC continue to experience substantially higher incidence than those without CMC/IC. Moreover, the IRRs comparing children with versus without risk conditions has generally increased over time, and are more pronounced in children ≥2 years of age. These findings underscore the need to evaluate and strengthen existing prevention strategies, including improving vaccine coverage rates and catch-up vaccination, to reduce IPD in children with risk conditions.

## Supplementary Material

IPD_IRs_by_risk_group_Supplemental_Tables_piag084

## Data Availability

The data that support the findings of this study are available from the Merative™ MarketScan® Commercial Database and the Multi-State Medicaid Database. These data were used under license for the current study, are not publicly available, and cannot be shared by the authors due to licensing restrictions. Access to the data may be obtained directly from Merative with appropriate permissions.

## Previous presentations

Part of the material in this manuscript was presented at the Pediatric Academic Societies (PAS) 2025 Meeting held April 24-28, 2025, in Honolulu, Hawaii as an oral presentation.
